# Development of a Deer Tick Virus Infection Model in C3H/HeJ Mice to Mimic Human Clinical Outcomes

**DOI:** 10.3390/v17081092

**Published:** 2025-08-07

**Authors:** Dakota N. Paine, Erin S. Reynolds, Charles E. Hart, Jessica Crooker, Saravanan Thangamani

**Affiliations:** 1 Department of Microbiology and Immunology, SUNY Upstate Medical University, Syracuse, NY 13210, USA; 2 SUNY Center for Vector-Borne Diseases, SUNY Upstate Medical University, Syracuse, NY 13210, USA; 3 Institute for Global Health and Translational Science, SUNY Upstate Medical University, Syracuse, NY 13210, USA; 4 Center for Global Health, Department of Internal Medicine, University of New Mexico Health Sciences Center, Albuquerque, NM 87131, USA; esreynolds@salud.unm.edu; 5 Defense Centers for Public Health–Aberdeen, Defense Health Agency-Public Health, Edgewood, MD 21040, USA; charles.e.hart120.mil@health.mil

**Keywords:** Arbovirus, Powassan virus, Deer tick virus, *Ixodes scapularis*

## Abstract

Deer tick virus (DTV) is a Tick-Borne Orthoflavivirus endemic to the United States, transmitted to humans through bites from the deer tick, *Ixodes scapularis*, which is also the primary vector of *Borrelia burgdorferi s.l.*, the causative agent of Lyme disease. Human infection with DTV can result in acute febrile illness followed by central nervous system complications, such as encephalitis and meningoencephalitis. Currently, there are mouse models established for investigating the pathogenesis and clinical outcomes of DTV that mimic human infections, but the strains of mice utilized are refractory to infection with *B. burgdorferi s.l.* Here, we describe the pathogenesis and clinical outcomes of DTV infection in C3H/HeJ mice. Neurological clinical signs, mortality, and weight loss were observed in all DTV-infected mice during the investigation. Infected animals demonstrated consistent viral infection in their organs. Additionally, neuropathology of brain sections indicated the presence of meningoencephalitis throughout the brain. This data, along with the clinical outcomes for the mice, indicates successful infection and showcases the neuroinvasive nature of the virus. This is the first study to identify C3H/HeJ mice as an appropriate model for DTV infection. As C3H/HeJ mice are already an established model for *B. burgdorferi s.l.* infection, this model could serve as an ideal system for investigating disease progression and pathogenesis of co-infections.

## 1. Introduction

Tick-borne diseases are increasingly becoming a significant public health burden worldwide. According to the CDC, the number of cases of tick-borne diseases has increased over the years [1]. Currently, 160 viruses worldwide are known to be tick-borne, spread primarily through the act of blood meal acquisition from a host [2]. The prevalence of viruses in relation to tick vectors allows them to cover a single DNA viral family, Asfarviridae, and eight total RNA viral families, being Orthomyxoviridae, Flaviviridae, Reoviridae, Rhabdoviridae, Nymaviridae, Nairoviridae, Phenuiviridae, and Peribunyaviridae [3]. Most tick-borne viruses cause severe human disease, manifesting as CNS disease or hemorrhagic fevers. These diseases may present clinically, allowing for detection, or may remain unnoticed in an asymptomatic state. An increase in vector-to-host contact potential is partially responsible for the rise in disease cases, as previously mentioned. In the United States, the rise in the Powassan virus (POWV) has been observed [4]. POWV is a member of the Orthoflaviviridae family and was initially isolated from the brain of a child suffering from a high fever and encephalitis [5]. There are two distinct lineages of POWV, named Powassan virus Lineage I and Powassan virus Lineage II. From 2004 to 2022 there were more than 290 cases of POWV in the United States, with over 250 of those cases being neuroinvasive and requiring hospitalization [6]. POWV-Lineage II, otherwise known as Deer tick virus (DTV) was first isolated from field-collected *Ixodes scapularis* that were circulating in Lyme disease-endemic areas [7]. Both lineages have similar replication stages, with slight changes in their primary vector. POWV mainly circulates in *Ixodes cookei* ticks which primarily feed on groundhogs, while DTV is transmitted by *Ixodes scapularis* that commonly feed on white-footed mice [8,9]. DTV shares about 84% nucleotide sequence similarity and 94% amino acid identity with POWV, while being serologically indistinguishable from one another [10,11]. Given those differences, recent work has shown that there are even pathological differences in the presentation of disease in animal models, including variations in frequency of specific neurological disease, where POWV presents with a skew towards paralysis and DTV presents primarily with rapid weight loss and seizures, in mice [12].

DTV infections in humans have been poorly described in terms of pathogenesis and neuroinvasion because they are often asymptomatic in healthy individuals and represent a much milder disease than POWV [13]. Infected individuals generally experience symptoms that range from mild to severe encephalitis or an unrecognizable asymptomatic infection. The primary medical condition for survivors of the disease is the significant neurological sequelae following viral clearance. There are no FDA-approved vaccines or treatments for DTV infection, which is a concern, as the current standard of care is symptom management.

There are currently several models established that effectively demonstrate the pathogenesis and clinical manifestations of both POWV lineages. These models play a crucial role in advancing knowledge that may lead to the development and evaluation of vaccines and therapeutics to combat infection. Most studies on POWV development utilize either BALB/c or C57BL/6 mice, as both strains exhibit febrile illness and comparable neurological manifestations when infected with POWV [14]. Both models are able to show varying levels of pathological outcomes that mimic outcomes in humans, and under laboratory conditions, provide excellent means of measuring febrile illness and presentation. In nature, *I. scapularis* commonly exhibits co-infection with multiple human pathogens, leading to a gap in our understanding of how pathogens may modulate disease transmission and severity [15]. Even more concerning is the number of *Ixodidae* ticks within this group that are co-infected with multiple pathogens, presenting the possibility of transmitting multiple diseases to a host in a single feeding. Several field collection studies have presented findings of ticks positive for multiple pathogens across several geographic areas where POWV is endemic [16,17]. The prevalence of co-infected tick feedings calls for models capable of presenting the means of infection for study and use.

In that vein, many animal models are used to study different tick-borne diseases, but few are acceptable models for *Borrelia burgdorferi s.l.* infection. In the past, a well described model that has been used was C3H/HeJ mice. These mice contain a random mutation in *Tlr4* making them resistant to LPS endotoxins, and extremely susceptible to various Gram-negative bacteria, making them useful for studies involving those pathogens [18,19]. This specific mutation, however, does not have direct evidence for impact in spirochete-based experimentation. In studies comparing disease presentation, C3H/HeJ mice were found to exhibit pronounced inflammatory arthritis as compared to other models, making them an excellent target for the Lyme disease causing agent [20]. In that regards, tick borne encephalitis virus (TBEV) has been successfully modeled in C3H/HeN mice [21]. This agent, primarily endemic to Europe and Asia, has been implicated in co-infections as well, specifically with *B. afzelii* [22]. Coinfection with *B. burgdorferi s.l.* has been shown to increase DTV viral burden in *I. scapularis* ticks [23]. Given that both *B. burgdorferi s.l.* and DTV are transmitted by the same vector, in the context of co-infection, this outlines a specific niche that is in dire need of being filled for model development. Our work outlines DTV infection in C3H/HeJ mice in order to be used as an effective model to investigate tick-borne coinfections, with a specificity towards DTV and *B. burgdorferi s.l.*

## 2. Materials and Methods

### 2.1. Ethics Statement

Animal experiments were conducted in Animal Biosafety Level 3 (ABSL3) facilities under SUNY Upstate Medical University Institutional Animal Care, and Use Committee approved protocol #499.

### 2.2. Virus

Deer-tick virus Spooner strain (DTV) was provided to us by the World Reference Center for Emerging Viruses and Arboviruses at the University of Texas Medical Branch (Galveston, TX, USA). The virus was initially passaged once in suckling mouse brains followed by six passages on Vero E6 cell ATCC (American Type Culture Collection). Previously, our DTV stocks were sequenced using next-generation sequencing to demonstrate that the stock was 99.84% identical to the DTV Spooner, Wisconsin isolate (GenBank: HM440560.1). There were three amino acid sequence differences in M (N213K), NS4B (S2368T), and NS5 (K2903R). Cells were cultured in Dulbecco’s Modified Eagle Medium (DMEM) supplemented with 10% heat-inactivated fetal bovine serum (FBS) and 1% penicillin/streptomycin and maintained at 37 °C and 5% CO_2_. Virus titer was determined by focus-forming assay as previously described [24].

### 2.3. Animals

Twelve-week-old female C3H/HeJ mice were purchased from The Jackson Laboratory (Bar Harbor, ME). Mice were group-housed, five per cage, in individually ventilated cages with HEPA-filtered supply and exhaust air. Food and water were provided ad libitum. Environmental conditions, including temperature, humidity, and the light-dark cycle, were monitored and maintained according to recommended settings [25]. Mice were acclimated for one week and study initiation occurred when animals were 13 weeks old.

All mice received a single footpad injection of 20 µL while under isoflurane anesthesia. Control mice received serum-free DMEM, and infected mice received 10^3^ FFU (Focus Forming Units) of DTV. Body weight measurements and detailed clinical observations were performed daily to assess animal appearance, behavior, respiration, and neurological signs of disease (Table 1). Mice were euthanized by CO_2_ inhalation followed by cervical dislocation when they reached the criteria for humane euthanasia or on Day 28. Submandibular blood collection was performed daily between 0 and 4 days post infection (dpi) using alternating cohorts of mice and alternating sites. Terminal blood was collected via cardiac puncture following euthanasia.

Tissues collected at necropsy were subdivided and stored in either Trizol or 10% Neutral Buffered Formalin (NBF). Blood was stored in Trizol LS. Samples stored in Trizol or Trizol LS were maintained in ABSL3 facilities for a minimum of 24 h to allow inactivation. Samples stored in 10% NBF were fixed for a minimum of 72 h with one change in NBF occurring 24 h after collection.

### 2.4. RNA Extractions and qRT PCR

As mice reached the terminal stage of disease, they were euthanized via CO_2_ asphyxiation followed by cervical dislocation and the following organs were harvested and stored in TRIzol Reagent (Invitrogen, Life Technologies, Carlsbad, CA) as instructed by the manufacturer’s recommendations: brain, heart, kidney, spleen, popliteal lymph node, and injection site. Harvested tissues were homogenized using sterile stainless-steel beads with a TissueLyser II (Qiagen) for 5 min at 30 Hz. Tissue and blood NA extractions were performed using a combination of TRIzol/TRIzol LS reagent and Qiagen RNeasy mini protocols, as we had previously demonstrated that a combination of these protocols works to inactivate the virus and yield high-quality RNA [26]. Chloroform was added to the tissue homogenate at the volume recommended by the TRIzol/TRIzol LS Reagent protocol. Samples were mixed by vigorous shaking for 20 s and incubated at room temperature for 3 min, following this, they were centrifuged at 12,000× *g* for 15 min at 4 °C. The upper aqueous layer was collected and mixed with one volume of 70% ethanol with pipetting. The mixture was then applied to a RNeasy Plus Mini column (Qiagen), and the kits designated spin protocol was followed. Total RNA was eluted by adding 50 µL of nuclease-free water to the column and spinning. RNA quantity and quality were measured with a DS-11+ spectrophotometer (Denovix).

Quantification of viral loads in the tissues was determined through quantitative reverse transcription real-time PCR (qRT PCR) using forward (5′-GCATGGTCGGATGAACAGAA-3′) and reverse (5′-CATTGGCCTTTCAGGTGTCT-3′) primers specific for the NS5 region of DTV (Integrated DNA Technologies, Coralville, IA) [22]. RNA was previously extracted from DTV stock of known titer that was used to infect mice for this study. Serial dilutions were previously made from the resulting RNA and a linear equation was generated by plotting cycle threshold (Ct) values of the standard curve against the log titer. Viral load Ct values from organ samples were determined by qRTPCR and converted to Log10 FFU equivalents using the linear equation from the standard curve, as described previously. In short, a standard amount of RNA had been added to each well of a Bio-Rad 96-well plate. 10 µM of qRTPCR forward and reverse primers specific for the DTV NS5 gene were added to each well and mixed with reagents from the iTaq Universal Probes One-Step Kit in a 20 µL total reaction volume (Bio-Rad, Hercules, CA). Plates were sealed and run on a CFX96 Real-Time System PCR platform (Bio-Rad, Hercules, CA) with the following cycling protocol: 10 min at 50 °C; 3 min at 95 °C; 15 s at 95 °C followed by 30 s at 60 °C for 45 cycles; and 81-cycle (+0.5 °C/cycle) 55–95 °C melt curve.

### 2.5. Histology

Following virus inactivation, tissues were removed from ABSL3 containment, and transferred to 70% EtOH and shipped to Histowiz, Inc. (Brooklyn, NY, USA) for paraffin embedding and sectioning. Tissues were sectioned into 5 µM sections and mounted onto positively charged glass slides. Hematoxylin and eosin staining was performed through Histowiz according to their established protocols. Slide interpretation was assessed according to the Histowiz in house pathologist. Briefly, Sagittal brain sections were examined in the rostro-caudal direction, including the olfactory bulb (OB), cerebral cortex (CTX), hippocampus (HPF), thalamus (TH), hypothalamus (HY), midbrain (MB), pons (P), medulla oblongata (MY) and cerebellum (CB—Purkinje cell, granular cell and molecular cell layers). Microscopic lesions, including microgliosis (MG), perivascular cuffing (PC), and/or neuronal necrosis (NN), were graded as—(absence of lesions); + (minimal); ++ (mild); +++ (marked). The presence of meningitis, encephalitis or meningoencephalitis was coded in the last column of the table as M, E or ME, respectively. Separate brain sections were subjected to RNA in situ hybridization for viral staining as described below.

### 2.6. RNA In Situ Hybridization

RNA in situ hybridization (RNAish) was performed using RNAscope 2.5 chromogenic assay for FFPE tissues (Advanced Cell Diagnostics) as described previously [27]. Briefly 5 um brain sections were deparaffinized and peroxidases were quenched using hydrogen peroxide for 10 min. The slides were boiled for 15 min in RNAscope Target Retrieval Solution and then incubated at 40 °C for 30 min with RNAscope Protease Plus reagent in a HybEZ™ Humidity Control Tray. The brains then were hybridized with a probe for Powassan virus (POWV) positive-sense RNA (catalog number 415641) and RNAscope Probe- Mm-Itgax-C2 (catalog number 311501-C2) followed by signal amplification and detection steps performed in accordance with the manufacturer. The POWV RNAscope probe is cross-reactive with DTV RNA, not distinguishing between POWV Lineage I and DTV Lineage II. The Mus musculus Duplex Ppib and Polr2a duplex probe were included as a positive control. Slides were counterstained with a 50% solution of Gills’ hematoxylin I. RNAscope stained brain sections were scanned digitally using a light microscope, and whole digital slide scans were examined by a board-certified veterinary pathologist contracted through HistoWiz.

## 3. Results

### 3.1. Clinical Observations Following Inoculation of DTV-Spooner

**Figure 1:**
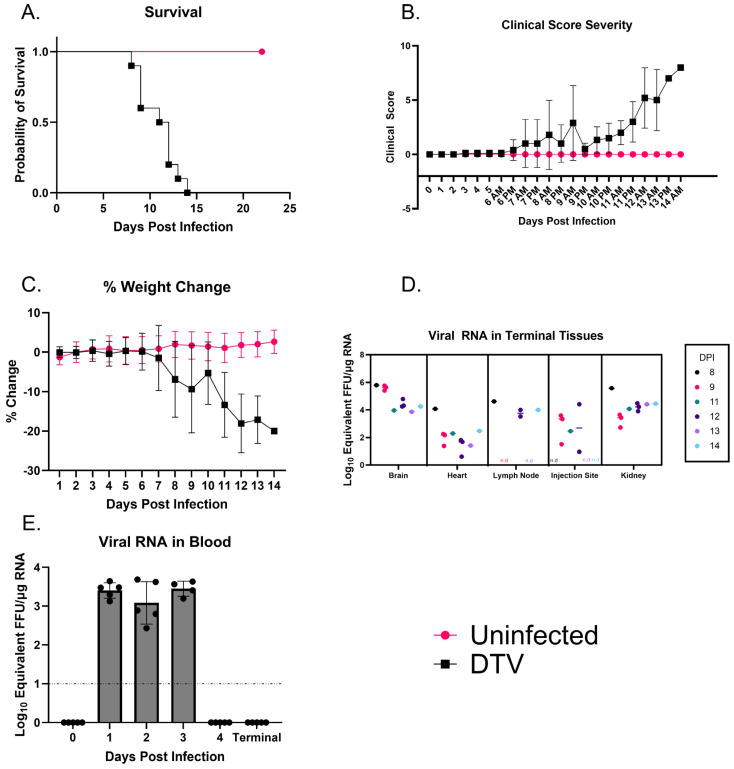
Clinical Observations and Detection of DTV in Infected Mice. (**A**). Kaplan–Meier survival curves for infected and uninfected cohorts (*n* = 10 per group). (**B**). Clinical scores for each mouse measured as a total sum at each day, up to full cohort mortality at 14 dpi. (**C**). Average weight loss across cohorts of mice. (**D**). Detection of DTV in organs harvested at the time of euthanasia and analyzed through qRTPCR. Viral load data is expressed using FFU equivalents/µg of RNA, post-normalization using a standard curve. Titers are plotted for each mouse tissue, separated further by date of euthanasia. The initials n.d. indicate not detectable for that specific time point/animal. The number of animals for each day is as follows: D8: 1, D9: 3, D11: 1, D12: 3, D13: 1, D14: 1. (**E**). Mice were bled via submandibular puncture on alternating days, and blood was analyzed for viremia using qRTPCR. Each symbol represents an individual mouse, and the bar represents mean values for each collection.

This C3H/HeJ model investigation comprises 10 DTV-infected mice and 10 uninfected mice. The mice that were infected with 10^3^ FFU of DTV all reached morbidity by day 14 (Figure 1A). Several of the mice reached morbidity by day 9, and one further reached morbidity by day 8. The mice showed no signs of disease except weight loss during the first 6–7 days of infection (Figure 1B). In most cases, the first observable sign of disease was an appearance-based change, typically associated with a weight loss of approximately 5–9%. Following this, reduced grooming and varying levels of appearance changes occurred as the days increased. As the infection progressed, there was a start of both respiratory and neurological changes, including shallow and labored breathing, reduced limb use, a weak grip, and ataxia. Around days 10–11, these minor disease manifestations of general malaise were followed up with neurological presentations and greater weight loss, resulting in >20% loss in mass (Figure 1C). The most impactful weight change occurred on average, after day 6, and the actual average weight change on day 13 was 17.11%. The neurological signs included the previously mentioned manifestations as well as tremors, paralysis, and seizures. A comprehensive list of clinical scores, based on a scoring system, is presented in Table 1.

### 3.2. Moribund C3H/HeJ Mice Show Consistent Viral Titers Across Major Organs

DTV infection was characterized using multiple organs of the infected mice (Figure 1D). Organs were harvested from each animal upon reaching the criteria for euthanasia, or 24 days after the start of the study, and qRTPCR was performed to look for the presence of DTV RNA. DTV RNA was detected in the brain, heart, lymph node, injection site, and kidney. RNA detected in the brains of each mouse was in a range of 7.7 × 10^3^ to 3.3 × 10^5^ FFU equivalents/µg RNA. The hearts of the mice contained a range of 4.1 to 1.1 × 10^4^ FFU equivalents/µg RNA. In the kidneys of all mice, there was viral detection ranging from 5.3 × 10^2^ to 3.8 × 10^5^ FFU equivalents/µg RNA. In the lymph nodes, RNA was detected at a range of 3.1 × 10^3^ to 4.1 × 10^4^ FFU equivalents/µg RNA. In the injection sites, RNA was detected in a range of 10 to 2.5 × 10^4^ FFU equivalents/µg RNA. All these mice succumbed to the infection. From 1 dpi through 3 dpi, DTV RNA was detected in the blood of all infected mice (Figure 1E). Viremia for the mice peaked around 3 dpi and had a sudden drop in detectable levels of 4 dpi. At 1 dpi, 2 dpi, and 3 dpi, the mean viral titers were found to be 2.5 × 10^3^, 1.2 × 10^3^, and 2.8 × 10^4^ FFU equivalents/µg RNA, respectively. Terminal blood showed minimal RNA detection that mostly remained undetectable in most animals (Figure 1E).

### 3.3. Histology and Neuropathology of DTV in the Brain

**Figure 2:**
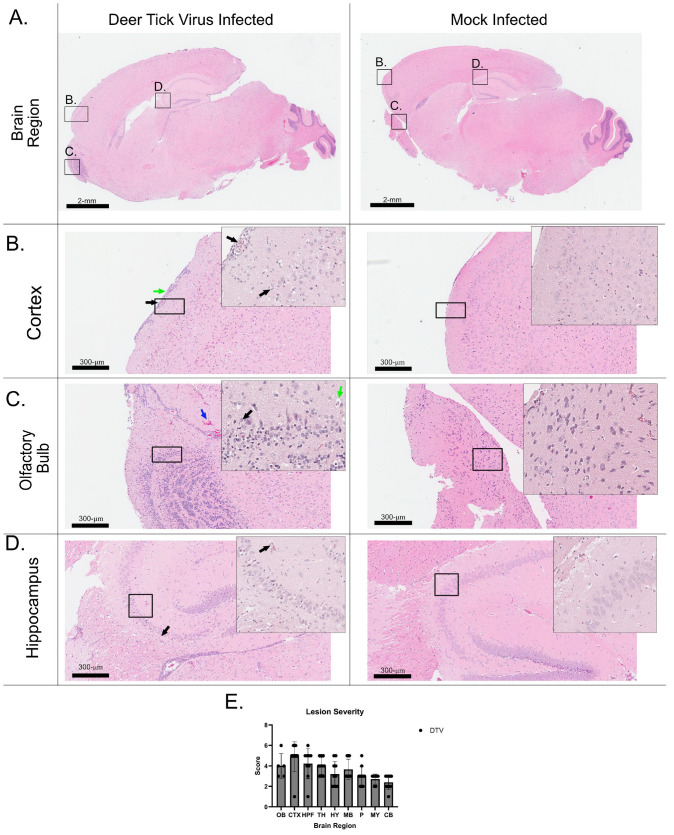
Hematoxylin and eosin-stained sections of the brain from DTV-infected mouse. This brain tissue was harvested from a moribund mouse at 9 dpi and H&E stained. (**A**) Whole brain sagittal cross-section; black boxes indicate the highlighted regions below (**B**) Isocortex of outer brain (green arrows showing infiltration of inflammatory cells into the subarachnoid space, and black arrows identifying vacuolation of neuropil and hyper-eosinophilic necrotic neurons). (**C**) Olfactory Bulb (Green arrows point to basophilic and shrunken neurons, and black arrows point to adjacent normal neurons, and blue arrows point to heavy red blood cell pooling). (**D**) Hippocampus (black arrows indicating nuclear pyknosis). (**E**) Lesion severity based on numeric injury scoring, grouped by brain region: olfactory bulb (OB), cerebral cortex (CTX), hippocampus (HPF), thalamus (TH), hypothalamus (HY), midbrain (MB), pons (P), medulla oblongata (MY) and cerebellum (CB—Purkinje cell, granular cell and molecular cell layers).

To assess the efficacy of active replication of DTV in the brain, H&E and a thorough histopathological analysis were performed (Figure 2). The analysis of neuropathology in the tissues was performed for 20 total mouse brains (10 control and 10 infected, Table 2). The distribution and severity of histologic lesions in different regions of the brain (olfactory bulb (Figure 2B), cerebral cortex (Figure 2C,E), hippocampus (Figure 2D), thalamus, hypothalamus, midbrain, pons, medulla oblongata, and cerebellum, comprising Purkinje cell, granular cell, and molecular cell layers) are presented according to semi-quantitative scores in Table 2 (Figure 2E). Microscopic lesions were absent in the brains of the ten uninfected mice.

All examined DTV-infected mice (10/10—animals #2001-2010) exhibited unequivocal, microscopic lesions characteristic of meningoencephalitis. These lesions were widespread and characterized by inflammation of the meninges and brain parenchyma, accompanied by microgliosis and perivascular cuffing, primarily composed of lymphocytes and monocytes. Neuronal necrosis and loss, accompanied by disruption of the adjacent neuropil (vacuolation), were observed in the most severely affected areas. Overall, microscopic lesions ranged from minimal to marked in severity in the affected regions (as indicated above) and tended to be more prominent in the cerebral cortex, brainstem (thalamus, hypothalamus, midbrain, pons, and medulla oblongata), cerebellum, and hippocampus (Table 2).

**Figure 3:**
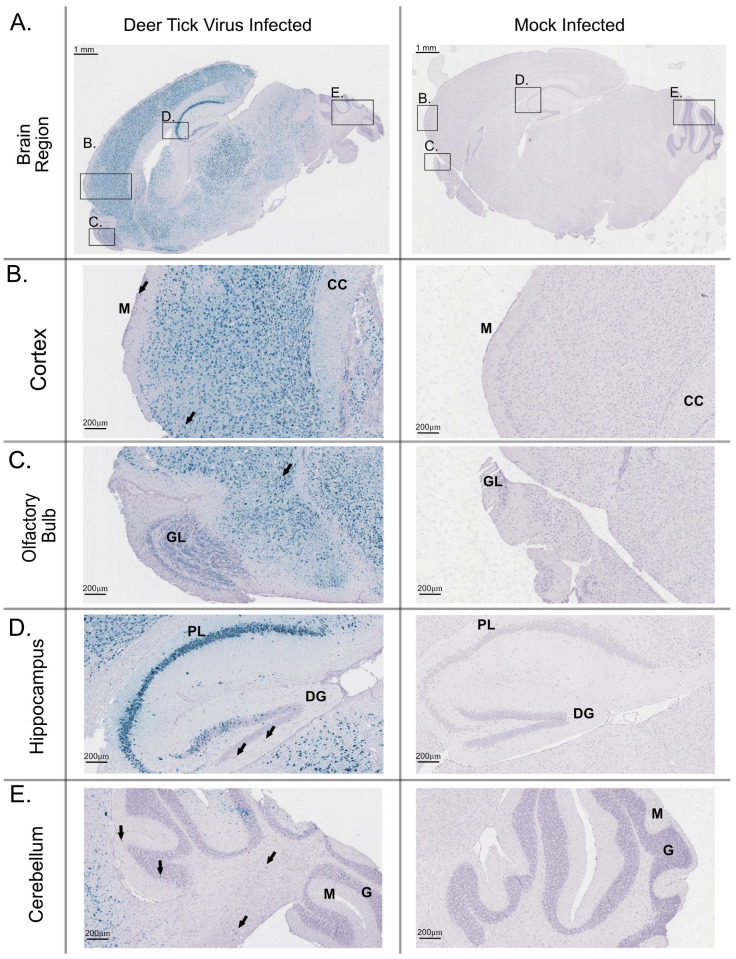
Distribution of positive-sense viral RNA in the brain of a mouse infected with DTV. This brain tissue was harvested from a moribund mouse at 9 dpi and stained for positive-sense POWV RNA (Teal stain), CD11b cell marker (magenta stain, black arrows), and counterstained with Hematoxylin. (**A**) Whole brain sagittal cross-section; black boxes indicate the highlighted regions below. Higher magnification of regions include the (**B**) cortex of the outer brain, including the inflamed meninges and corpus collosum (black arrows indicate CD11b+ signal, M: meninges, CC: corpus callosum) (**C**) olfactory bulb with the frontal cortex region (black arrows indicate CD11b+ signal, GL: granular layer), (**D**) hippocampus with pyramidal neuron layer and the dentate gyrus (black arrows indicate CD11b+ signal, PL: pyramidal layer, DG: dentate gyrus), and (**E**) cerebellum (black arrows indicate CD11b+ signal, M: molecular layer, G: granular layer).

RNA in situ hybridization was performed on a section of each brain that underwent H&E staining to detect viral RNA (Figure 3). In all mouse brains, a large amount of positive-sense genomic DTV RNA was detected. In various regions of the brain, including the olfactory bulb (Figure 3B), the isocortex region (Figure 3C), the hippocampus (Figure 3D), and the cerebellum (Figure 3E) positive-sense viral RNA was detected, ranging from low amounts of dot signal to an intense, numerous dot signal. The DTV signal was detected at higher intensities in regions such as the cortex, olfactory bulb, and hippocampus, while lower intensity regions included the hind brain and cerebellum. The localization and intensity of staining is consistent with the literature associated with these infections.

## 4. Discussion

Throughout North America, DTV is spread by tick vectors, including Ixodes sp. This species is known to spread a variety of human-relevant pathogens that are associated with poor outcomes [1,17]. Due to the increased effects of global warming, there has been a shift in the geographic distribution of viral vectors, such as ticks [28,29]. This increase in vector spread can be correlated to the increase in the diseases that they cause. Notably, well-known pathogens, such as those causing Lyme disease and anaplasmosis, have been increasing steadily over the past several years. Even more so, the number of cases of clinically presented POWV/DTV infection, have been seen to increase steadily as well [6,30].

Based on the analysis of the data, we suggest that the C3H/HeJ mouse model is suitable for modeling DTV infection. The age of 13 weeks old was selected based on previous work performed showing varying susceptibility to POWV infections in different animal models [31]. Additionally, the route of infection and the inoculum dose are characterized based on other animal model papers using dose responses via footpad inoculation [26]. We observed a median value of 6.3 × 10^5^ equivalent FFU/µg RNA in the brain, as well as similar levels at the injection site and kidneys of each mouse. Additionally, with the levels of virus detection in the blood peaking between 1 and 3 dpi, we show that the virus is successfully infecting the brain, as well as causing viremia, on those days. The weights of the mice decreased at an increased rate when they were infected with DTV when compared to the control uninfected cohort, giving a clear indication that mice were succumbing to infection. Between the clinical scoring and survival rates, there was more than sufficient data to suggest that these mice are appropriately modeling DTV infection in a manner comparable to that of other models established for DTV, such as BALB/c mouse models. Additionally, these mice may be a suitable model for POWV (Lineage I) as well, based on the two lineages’ similarities [12,26].

When scored clinically, the study mice all became moribund by 14 days post-infection (dpi), with most manifestations starting around 9 dpi. This is in line with previous data showing that DTV infection shows clinical disease starting 5–6 days post inoculation and morbidity occurring 9 dpi [32]. Given that the average time to euthanasia occurred at 11 dpi, these mice followed the model as outlined by previous animal models [12,33].

This study utilized a strain of mice that had not been used for DTV or POWV before. In prior experiments, BALB/c and C57BL/6 mice have been identified as suitable models for disease progression, as they exhibit characteristic signs of disease markers similar to those observed in human clinical manifestations [26,34]. While these established models have been shown to be efficacious in representing clinical outcome of single infections, none have been shown to be useful for *B. burgdorferi s.l.* infection to measure and represent patient clinical outcome. BALB/c mice are shown to only react mildly to an infection, in a specifically non-lethal model and C57BL/6 mice are mostly resistant to infection [35,36]. The use of C3H/HeJ, as outlined in this study, allows for the consideration of interesting co-infections that occur with DTV infection. Future investigations should aim to clarify the true dynamics of co-infection between Borrelia burgdorferi s.l. and Deer Tick Virus (DTV), especially focusing on the role of vector-based transmission. Since DTV is highly lethal in standard murine models, using vector-mediated infection methods might offer a more realistic ecological perspective for studying these interactions. Additionally, previous studies showing different pathogenic outcomes between Powassan virus (POWV) and DTV imply the potential for varied co-infection dynamics with *B. burgdorferi s.l.* Therefore, upcoming research should focus on understanding the specific interactions between each viral agent and *B. burgdorferi s.l.*, rather than treating POWV and DTV as interchangeable.

## 5. Conclusions

In conclusion, we have developed a small-animal model in C3H/HeJ mice for DTV pathogenesis that imitates the development of disease in humans. All mice used in the study were susceptible to the infection, and all mice developed neurological signs of the disease. Results from this study, including the rapid onset of neurological and behavioral disease manifestations, meningoencephalitis characterized by microscopic lesions and parenchymal cuffing, and viral load dissemination throughout the brain and other body organs, all point to the successful neurotropism of DTV in this model. Future work is required to further study the effects of different virus titers used for infection, which will be utilized in model comparison studies. Research has shown that POWV infection in ticks, when combined with a co-infection of *B. burgdorferi s.l.*, leads to an increase in replication and dissemination, making this model useful for the future study of coinfection of these two pathogens [23]. Additionally, this model design can assist in the validation and development of future therapeutics and vaccine candidates for DTV.

## Figures and Tables

**Table 1:** Scoring chart for clinical manifestations used in observational portions of in vivo disease progression. Signs with an “*” indicate criteria for immediate humane euthanasia.

Parameter	Degree of Parameter	Possible Score
Appearance	Normal (smooth coat, eyes/nose clear), weight loss < 4.99%	0
	Reduced grooming, weight loss between 5 and 9.99%, slightly ruffled	1
	Ruffled coat, ocular/nasal discharge, eye(s) partially closed, warm to touch, weight loss between 10 and 19.99%	2
	No grooming, eye(s) closed, hunched posture, pale, cold to touch, weight loss > 20% total body weight *	3
Neurological Signs of Disease	Normal	0
	Weak grip, reduced limb usage	1
	Paresis, ataxia, tremors, head tilt	2
	Total paralysis *, seizures *, unresponsive *, loss of righting reflex	3
Provoked Behavior	Normal	0
	Subdued but normal when stimulated	1
	Subdued even when stimulated, lethargic	2
	Unresponsive when stimulated *, moribund *, prostrate *	3
Respiration	Normal	0
	Rapid, shallow	1
	Diaphragmatic, labored	2
	Gasping *	3
Cumulative Score	Score of 0–5 = No intervention	
	Score of >6 = More frequent monitoring	
	Score of 8–10 = Contact PI and/or DLAR Veterinarian	
	Score of ≥11 = Euthanasia	

**Table 2:** Histopathological analysis of 10 DTV-infected animals. Microscopic lesions, including microgliosis (MG), perivascular cuffing (PC), and/or neuronal necrosis (NN), were graded as—(absence of lesions); + (minimal); ++ (mild); +++ (marked) The presence of meningitis, encephalitis or meningoencephalitis: coded in the last column of the table as M, E or ME, respectively. NP = region not present on the slide. OB = olfactory bulb, CTX = cerebral cortex, HPF = hippocampal formation, TH = thalamus, HY = hypothalamus, MB = midbrain, P = pons, MY = medulla, CB = cerebellum.

Sample ID	OB	CTX	HPF	TH	HY	MB	P	MY	CB			Meningitis/Encephalitis
									Purkinje Cells	Granule Cells	Molecular Cells	
2001	NP	+	-	NP	NP	+ MG, PC,	+ MG	NP	+ NN	+ NN	++	ME
		MG,				NN					MG	
		PC,										
		NN										
2002	+	++	++	+ MG,	++	+ MG, PC	+ MG	+ MG, PC	-	+ NN	+ MG	ME
	MG, NN	MG,	MG,	PC,	MG,							
		PC,	PC,	NN	PC,							
		NN	NN		NN							
2003	++	+++ MG,	++	++	++	+ +	+	NP	+ NN	+ NN	+ MG	ME
	MG, NN	PC,	MG,	MG,	MG,	MG,	MG,					
		NN	PC,	PC,	PC,	PC,	PC,					
			NN	NN	NN	NN	NN					
2004	+	++	++	++	++	++	+	+ MG, PC	-	+	+ MG	ME
	MG, PC	MG, PC, NN	MG, PC, NN	MG, PC, NN	MG, PC	MG, PC, NN	MG, PC			NN		
2005	NP	++	+ MG, PC	+ MG, PC	+ MG, PC	+ MG, PC	+	+ MG, PC	-	+ NN	+ MG	ME
		MG,					MG, PC					
		PC,										
		NN										
2006	NP	+++ MG,	+++ MG,	++	++	+ +	++	+ MG, PC	+ NN	+ NN	+ MG	ME
		PC,	PC,	MG,	MG, PC	MG,	MG,					
		NN	NN	PC,		PC,	PC,					
				NN		NN	NN					
2007	-	++	++	+ MG, PC	+ MG, PC	+ MG, PC	+	+ MG	+ NN	+ NN	++ MG	ME
		MG,	MG, PC				MG, PC					
		PC,										
		NN										
2008	-	+++	-	++	+ MG	+	+	+ MG, PC	+ NN	+ NN	+ MG	ME
		MG,		MG, PC		MG, PC	MG, PC					
		PC,										
		NN										
2009	NP	+++	++	++	+ MG, PC	+ MG	+	+ MG, PC	-	-	+ MG	ME
		MG,	MG,	MG,			MG, PC					
		PC,	PC,	PC,								
		NN	NN	NN								
2010	NP	++	+ MG, PC,	++	+ MG	+	+ MG	NP	-	+ NN	+ MG	ME
		MG,	NN	MG, PC		MG, PC						
		PC,										
		NN										

## Data Availability

All relevant data are within the manuscript.

## Abbreviations

The following abbreviations are used in this manuscript:

POWV	Powassan Virus
DTV	Deer Tick Virus
FDA	Food and Drug Administration
ABSL3	Animal Biosafety Level 3
DMEM	Dulbecco’s Modified Essential Media
FFU	Focus Forming Units
qRTPCR	Quantitative Real-Time Polymerase Chain Reaction
FFPE	Formalin Fixed Paraffin Embedded
H&E	Hematoxylin and Eosin
RNAish	RNA-In Situ Hybridization
dpi	Days Post Infection
